# Gastroesophageal reflux related changes after sleeve gastrectomy and sleeve gastrectomy with fundoplication: A retrospective single center study

**DOI:** 10.3389/fendo.2022.1041889

**Published:** 2022-11-18

**Authors:** Aikebaier Aili, Maimaitiaili Maimaitiming, Pierdiwasi Maimaitiyusufu, Yusujiang Tusuntuoheti, Xin Li, Jianyu Cui, Kelimu Abudureyimu

**Affiliations:** ^1^ Department of Minimally Invasive Surgery, Hernias and Abdominal Wall Surgery, People's Hospital of Xinjiang Uygur Autonomous Region, Urumqi, Xinjiang Uygur Autonomous Region, China; ^2^ Xinjiang Clinical Research Center for Gastroesophageal Reflux Disease and Bariatric Metabolic Surgery, Urumqi, Xinjiang Uygur Autonomous Region, China; ^3^ Research Institute of General and Minimally Invasive Surgery, Urumqi, Xinjiang Uygur Autonomous Region, China; ^4^ The Graduate Student Institute of Xinjiang Medical University, Urumqi, Xinjiang Uygur Autonomous Region, China

**Keywords:** gastroesophageal reflux disease (GERD), laparoscopic sleeve gastrectomy (LSG), laparoscopic sleeve gastrectomy with fundoplication, *de novo* GERD after sleeve gastrectomy, GERD after bariatric surgery

## Abstract

**Background:**

The worsening of gastroesophageal reflux disease (GERD) and “*de novo*” GERD after laparoscopic sleeve gastrectomy (LSG) is a major concern as it affects the patient’s quality of life; the incidence of GERD after LSG is up to 35%. Laparoscopic sleeve gastrectomy with fundoplication (LSGFD) is a new procedure which is considered to be better for patients with morbid obesity and GERD, but there is a lack of objective evidence to support this statement. This study aimed to assess the effectiveness, safety, and results of LSG and LSGFD on patients who were morbidly obese with or without GERD over an average of 34 months follow-up.

**Methods:**

Fifty-six patients who were classified as obese underwent surgery from January 2018 to January 2020. Patients who were obese and did not have GERD underwent LSG and patients who were obese and did have GERD underwent LSFGD. The minimum follow-up time was 22 months and there were 11 cases lost during the follow-up period. We analyzed the short-term complications and medium-term results in terms of weight loss, incidence of *de novo* GERD/resolution of GERD, and remission of co-morbidities with follow-up.

**Results:**

A total of 45 patients completed the follow-up and a questionnaire-based evaluation (GERD-Q), of whom 23 patients underwent LSG and 22 patients underwent LSGFD. We had 1 case of leak after LSGFD.No medium or long- term complications. The patient’s weight decreased from an average of 111.6 ± 11.8 Kg to 79.8 ± 12.2 Kg (*P* = 0.000) after LSG and from 104.3 ± 17.0 Kg to 73.7 ± 13.1 Kg (*P* = 0.000) after LSGFD. The GERD-Q scores increased from 6.70 ± 0.5 to 7.26 ± 1.7 (P = 0.016) after LSG and decreased from 8.86 ± 1.3 to 6.45 ± 0.8 (*P* = 0.0004) after LSGFD. The incidence of *de novo* GERD after LSG was 12 (52.2%) at the 12 month follow-up and 7 (30.4%) at the mean 34 (22–48) month follow-up. The remission of reflux symptoms, for patients who underwent LSGFD, was seen in 19 (86.4%) of 22 patients at 12 months and 20 (90.9%) of 22 patients at the mean 34 (22-48) month follow-up. The two groups did not have any significant difference in the effect of weight reduction and comorbidity resolution.

**Conclusion:**

The incidence of *de novo* GERD after LSG is high,LSG resulted in the same weight loss and comorbidity resolution as LSGFD, in patients who are morbidly obese and experience GERD, and LFDSG prevent the occurrence and development of GERD, combination of LSG with fundoplication (LSGFD) is a feasible and safe procedure with good postoperative results,which worthy of further clinical application.

## Introduction

Obesity has become a serious public health problem (1). Recent statistics show that overweight/obesity continues to rise globally, with more than 2 billion people being overweight and accounting for approximately 30% of the world population (2). Gastroesophageal reflux disease (GERD), a known obesity-related complication, is a condition that occurs when reflux of stomach contents causes troublesome symptoms such as heart burn, regurgitation and chest pain (3). According to a meta-analysis, the global pooled prevalence of weekly gastroesophageal reflux symptoms is roughly 13% (4). Interestingly, the incidence of GERD in morbidly obese patient is up to 73% (5).

Bariatric surgery is considered the most effective therapy for morbid obesity at present and sleeve gastrectomy (SG) is now the most widely performed bariatric procedure (6). A large number of studies have reported good overall results with regards to surgical safety, resolution of obesity-related morbidities, and medium-term results for SG (7–9). However, SG is associated with a high incidence of GERD in long-term follow-up (10). For this reason, SG is not recommended for morbid obesity with GERD. Roux-en-Y gastric bypass is the best option for patients with obesity and GERD, but the long-term follow up to gastric Y-bypass outcomes showed that the treatment efficacy of gastric bypass on reflux symptoms might have been overestimated (11). Recently, the invention of a new surgical approach that reduces the incidence of postoperative GERD has gained good overall short-term results (12, 13). This study aimed to assess the effectiveness, safety, and results of laparoscopic SG (LSG) and LSG combined with fundoplication (LSGFD) on patients who were morbidly obese with or without GERD over an average of 34 months follow-up.

## Materials and methods

### Ethics statement

This study involved human participants and was reviewed and approved by the Independent Ethics Committee of People’s Hospital of Xinjiang Uygur Autonomous Region December 2017(NO.2017-94-XJS). We have received patients consent for participation in this study (operation consent, new technology consent etc.).

### Data sources

**Figure 1:**
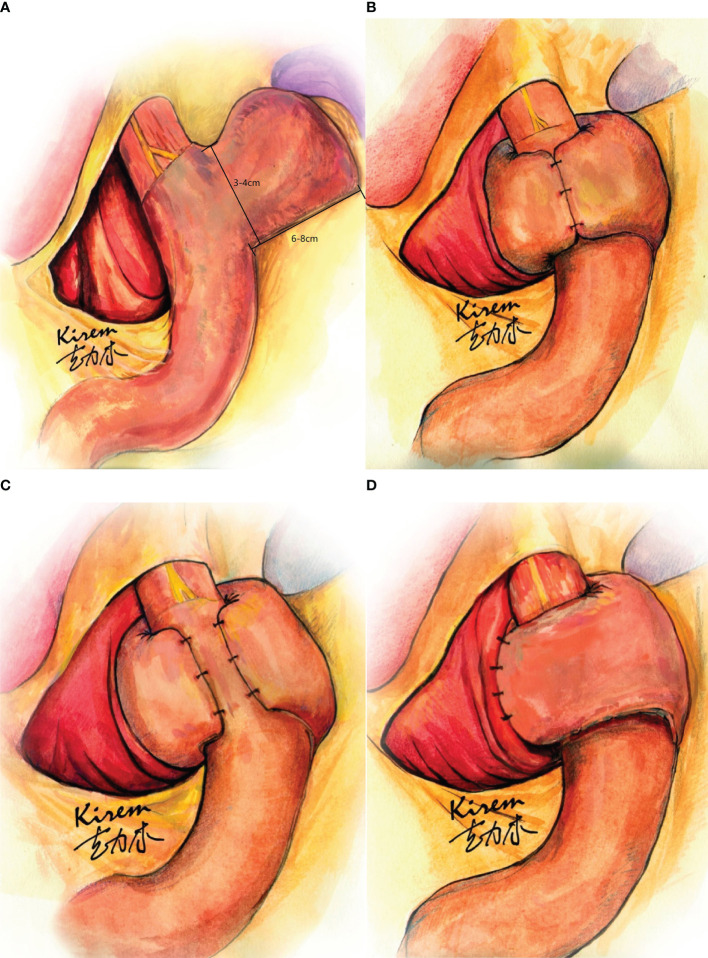
Laparoscopic sleeve gastrectomy with fundoplication (LSGFD). **(A)** Sleeve gastrectomy with preserved fin shaped fundus; **(B)** Sleeve gastrectomy with Nissen fundoplication; **(C)** sleeve gastrectomy with Toupet fundoplication; **(D)** Sleeve gastrectomy with Dor fundoplication.

This retrospective study analyzed data from Fifty-six patients who either underwent LSG or the modified anti-reflux fundoplication procedure (LSGFD) from January 2018 to January 2020. All the patients were classified as morbidly obese and were suitable candidates for surgery following the recommendations of the China Society for Bariatric and Metabolic Surgery. All the procedures were performed at the same hospital by a single surgeon who has extensive experience in laparoscopic bariatric and GERD surgery. The minimum follow-up time was 22 months and there were 11 cases (5 cases of LSG and 6 cases of LSGFD)lost during the follow-up period. Of the 45 bariatric surgical procedures performed on patients, 23 were LSG procedures for patients who did not have GERD (defined as a DeMeester score of <14.7 or GERD questionnaire [GERD-Q] score of < 8) and 22 were LSGFD (**Figure 1**) for patients who experienced GERD. All patients were examined preoperative for the severity GERD by means of the GERD-Q, esophageal 24-hour multichannel intraluminal impedance, a pH monitoring study, and an upper gastrointestinal scope to detect reflux disease signs, esophagitis (classified according to the Los Angeles classification), and Barrett’s esophagus. The preoperative characteristics of the study population are summarized in **Table 1**. All patients underwent a multidisciplinary evaluation preoperative by a psychologist, dietician, and anesthesiologist; instrumental evaluation included polysomnography, abdominal ultrasound, and upper endoscopy. Informed consent for surgery was obtained from each patient preoperatively. The primary outcome of the study was the weight loss parameters (% excess weight loss [EWL] and % total weight loss [TWL]) at a mean of 34 (22–48) months after surgery. The secondary outcomes include changes in the GERD-Q, the incidence of *de novo* GERD (defined as a GERD-Q score of ≥ 8 at 6 months postoperatively), and the incidence of the resolution of GERD (defined as a GERD-Q score of < 8 at 6 months postoperatively). The percentage of EWL and TWL were calculated as follows:

**Table 1:** Patient’s demographics and preoperative evaluations.

Parameters	LSG (n=23)	LSGFD (n=22)	P
Sex, % of females	73.9	77.3	0.524
Age (yrs), mean ± SD (range)	36.3± 10.7 (20-57)	37.9 ± 9.1 (21-56)	0.592
Hypertension, n (%)	7 (30.4%)	6 (27.3%)	0.815
Sleep apnea syndrome, n (%)	16 (69.6%)	12 (54.5%)	0.299
Diabetes Mellitus Type II, n (%)	7 (30.4%)	6 (27.3%)	0.815
Weight (Kg), mean ± SD (range)	111.6 ± 11.8 (92-135)	104.3 ± 17.0 (79.5-143)	0.098
BMI (Kg/m^2^), mean ± SD (range)	39.6 ± 4.5 (33.4-50.8)	38.4 ± 5.7 (31.1-50.2)	0.442

SD, standard deviation; LSG, Laparoscopic sleeve gastrectomy; LSGFD, Laparoscopic sleeve gastrectomy with fundoplication; BMI, body mass index; EWL, excess weight loss; TWL, total weight loss.

%EWL = 100% × (initial BMI - follow up BMI)/(initial BMI - 23)

%TWL = 100% × (initial BMI - follow up BMI)/(initial BMI - 0)

### Surgical technique

Laparoscopic techniques were used for both of the procedures included in this study.

The procedure for LSG was as follows: After effective general anesthesia was obtained, routine disinfection of the surgical field was performed, and four trocars were introduced. The surgeon stood at the patient’s right side. A 10-mm trocar was inserted 3 cm above the umbilicus (this trocar was used for the insertion of a 30° angled optic that guaranteed complete intraperitoneal exploration); a second 5 or 10-mm trocar was inserted 4 cm below the costal margin in the left midclavicular line; a third trocar of 5-mm was inserted 2 cm below the costal margin in the right anterior axillary line; and a fourth trocar of 12 mm was inserted at the umbilical level in the right midclavicular line. A 2 mm skin incision was made under the xiphoid and a self-made “S”-shaped thick iron wire liver lobe retractor was inserted (this retractor was used to expose the lesser curvature of the stomach around the esophageal cardia). The first surgical step was the dissociation of the greater curvature, fundus, and posterior wall of the stomach with the ultrasonic scalpel, starting 3 cm from the pylorus and ending with the dissociation of the fundus. The esophagus was intubated with a 36-F bougie which was advanced to the side of the lesser curvature of the stomach. If a hiatal hernia was present, it was repaired. The next surgical step was sleeve gastrectomy which started 3 cm from the pylorus and ended with the resection of most of the greater gastric curvature and the fundus. After it was confirmed that there was no air leakage at the gastric incision margin, the anastomotic margin of the residual stomach was continuously sutured with a surgical barbed suture to prevent bleeding and leakage.

The procedure for LSGFD was as follows: The difference between this surgery and LSG was that a small, fin-shaped part of the fundus was preserved during gastric resection (**Figure 1A**). To ensure the reliability of the weight loss effect, the retained gastric fundus should not be made too large; approximately 3–4 cm in width and 6–8 cm in length, or enough for appropriate folding (due to individual differences in esophageal thickness), and the appearance is similar to that of a fin. The residual gastric cavity was checked for margin leakage *via* gastric gas injection, and the residual gastric suture was continuously stitched to strengthen the absorbable suture and prevent postoperative bleeding and fistula formation. A Nissen or Toupet or Dor fundoplication was performed, depending upon the length of fundus preserved after the SG (**Figures 1B–D**).

### Statistical analysis

Continuous variables with a normal distribution are expressed as the mean ± standard deviation. Categorical variables are expressed with the use of frequencies. To compare the preoperative and postoperative parameters for each surgery, we used the X^2^ test for categorical variables and Student’s paired t test for continuous data. The independent samples test was used to compare the parameters between LSG and LSGFD. A P value of 0.05 was considered statistically significant. Statistical analyses were performed using SPSS version 26.0 (IBM Corp., Armonk, N.Y., USA).

## Results

A total of 45 patients completed the preoperative evaluation, of whom 23 patients underwent LSG while 22 patients underwent LSGFD. A questionnaire-based evaluation (GERD-Q) was completed by trained resident physician. The patient’s basic demographic characteristics and improvement of weight-related parameters are shown in **Table 1**. There were no significant differences in these parameters between the groups.

**Table 2** highlights the preoperative and postoperative data. Only four trocars were used for all the procedures, and additional trocars were not necessary. No intraoperative complications were reported. The perioperative and postoperative mortality rate was 0%. The mean length of hospital stay was 7.3 ± 2.1 (range 4–11) days for LSG and 6.5 ± 1.7 (range 3–11) days for LSGFD, there was no significant difference between the groups. There were no reported medium or long- term complications following LSG and LSGFD. Major complications (1(4.3%) LSG vs 2(9.0%) LSGFD): There was one (4.5%) case of leak after LSGFD. Bleeding was diagnosed in one (4.3%) patient in LSG group versus one (4.5%) patient in LSGFD. Minor complications (5(21.7%) LSG vs 5(22.7%) LSGFD: Nausea & vomiting diagnosed in 3 (13.0%) patients in LSG and 4 (18.2%) in LSGFD. Wound infections diagnosed in 2(8.7%)patients in LSG and 1 (4.5%) in LSGFD.

**Table 2:** Perioperative and postoperative data.

Surgical procedure	LSG (n=23)	LSGFD (n=22)	P
**Perioperative course**			
Trocars, n, mean ± SD (range)	4 ± 0 (4)	4 ± 0 (4)	
Conversion, n (%)	0 (0)	0 (0)	
**Postoperative course**			
Length of hospital stay, d, mean ± SD (range)	7.3 ± 2.1 (4-11)	6.5 ± 1.7 (3-11)	0.150
Major complications (%)	1 (4.3%)	2 (9.0%)	0.483
Bleeding (%)	1 (4.3%)	1 (4.5%)	0.744
Leak (X-rays,endoscopy), n (%)	0 (0)	1 (4.5%)	0.489
Minor complications (%)	5 (21.7%)	5 (22.7%)	0.609
Nausea &vomit(%)	3 (13.0%)	4 (18.2%)	0.474
Wound infections (%)	2 (8.7%)	1 (4.5%)	0.517
Reoperation, n (%)	0 (0)	0 (0)	
30-d readmission, n (%)	0 (0)	0 (0)	
Deaths, n (%)	0 (0)	0 (0)	

SD, standard deviation; LSG, Laparoscopic sleeve gastrectomy; LSGFD, Laparoscopic sleeve gastrectomy with fundoplication.

**Table 3** shows the changes in weight related evaluation after LSG and LSGFD, reflected as a significant decrease in weight from 111.6 ± 11.8 Kg to 79.8 ± 12.2 Kg after LSG, and from 104.3 ± 17.0 Kg to 73.7 ± 13.1 Kg after LSGFD.

**Table 3:** Comparison of preoperative and postoperative weight related evaluation after LSG and LSGFD.

	LSG (n=23)			LSGFD (n=22)		
	Preoperative	Postoperative	*P* value	Preoperative	Postoperative	*P* value
Weight (Kg), mean ± SD	111.6 ± 11.8	79.8 ± 12.2	0.000*	104.3 ± 17.0	73.7 ± 13.1	0.000*
BMI(Kg/m2), mean ± SD	39.6 ± 4.5	28.3 ± 4.2	0.000*	38.4 ± 5.7	27.3 ± 5.2	0.000*

LSG, Laparoscopic sleeve gastrectomy; LSGFD, Laparoscopic sleeve gastrectomy with fundoplication; BMI, body mass index. *Statistically significant.

**Table 4** shows the changes in GERD symptoms based on GERD-Q scores. Interestingly, the GERD-Q scores decreased after LSGFD (from 8.86 ± 1.3 to 6.45 ± 0.8), while there was an increase after LSG(from 6.70 ± 0.5 to 7.26 ± 1.7). The scores indicated a statistical significance in both groups when compared with the preoperative and postoperative status.

**Table 4 (A):** Changes in questionnaire scores after LSG and LSGFD.

Questionnaire	LSG (n=23)			LSGFD (n=22)		
	Preoperative	Postoperative	*P* value	Preoperative	Postoperative	*P* value
GERD-Q	6.70 ± 0.5	7.26 ± 1.7	0.016*	8.86 ± 1.3	6.45 ± 0.8	0.0004*

LSG, Laparoscopic sleeve gastrectomy; LSGFD, Laparoscopic sleeve gastrectomy with fundoplication; GERD-Q, gastroesophageal reflux disease questionnaire; *Statistically significant.

**Table 4 (B):** Comparison of preoperative and postoperative gastroesophageal reflux disease questionnaire (GERD-Q) scores after LSG and LSGFD.

GERD-Q	LSG (n=23)	LSGFD (n=22)	*P* value
Preoperative	6.70 ± 0.5	8.86 ± 1.3	0.000*
Postoperative	7.26 ± 1.7	6.45 ± 0.8	0.045*

LSG, Laparoscopic sleeve gastrectomy; LSGFD, Laparoscopic sleeve gastrectomy with fundoplication; *Statistically significant.

**Table 5** demonstrates the follow-up results. The EWL percent (EWL%) at 6, 12, and the mean of 34 (22–48) months was 55.2 ± 21.3%, 76.6 ± 21.6%, and 70.2 ± 21.8%, respectively, after LSG and 53.4 ± 27.8%, 83.3 ± 28.5%, and 77.9 ± 31.3%, respectively, after LSGFD. The TWL percent (TWL%) at 6, 12, and the mean of 34 (22–48) months was 22.1 ± 7.6%, 31.0 ± 8.2%, and 28.5 ± 8.1%, respectively, after LSG and 20.0 ± 9.3%, 31.2 ± 8.0%, and 29.0 ± 9.2%, respectively, after LSGFD. The two groups did not have any significant difference in these parameters, P > 0.05. The incidence of de novo GERD (defined as a GERD-Q score ≥ 8) after LSG was 12(52.2%) at the 12 month and 7 ( 30.4% ) at the mean 34 (22–48) month follow-up. Remission of reflux symptoms (defined as a GERD-Q score < 8) was seen in 19 (86.4%) of 22 patients at 12 months and 20 (90.9%) of 22 patients at mean 34 (22–48) months after LSGFD.

**Table 5:** Results of follow-up.

Items	6 months			12 months			24+ months		
	LSG	LSGFD	p	LSG	LSGFD	p	LSG	LSGFD	p
Weight (Kg), mean ± SD	87.0 ± 12.9	83.0 ± 13.5	0.319	76.9 ± 11.5	71.4 ± 12.1	0.127	79.8 ± 12.2	73.7 ± 13.1	0.113
BMI (Kg/m2), mean ± SD	30.9 ± 5.1	30.8 ± 5.9	0.931	27.3 ± 4.4	23.6 ± 4.2	0.447	28.3 ± 4.2	27.3 ± 5.2	0.470
EWL (%), mean ± SD	55.2 ± 21.3	53.4 ± 27.8	0.815	76.6 ± 21.6	83.3 ± 28.5	0.371	70.2 ± 21.8	77.9 ± 31.3	0.342
TWL (%), mean ± SD	22.1 ± 7.6	20.0 ± 9.3	0.401	31.0 ± 8.2	31.2 ± 8.0	0.925	28.5 ± 8.1	29.0 ± 9.2	0.847
Other comorbidities									
Sleep apnea, n (%)	7 (30.4%)	6 (27.3%)	0.815	2 (8.7%)	3 (13.6%)	0.635	2 (8.7%)	3 (13.6%)	0.560
Hypertension, n (%)	3 (13.0%)	4 (18.2%)	0.634	2 (8.7%)	2 (9.1%)	0.936	2 (8.7%)	2 (9.1%)	0.936
Diabetes, n (%)	1 (4.3%)	2 (9.1%)	0.524	0 (0%)	1(4.5%)	0.301	0 (0%)	0 (0%)	–

LSG, Laparoscopic sleeve gastrectomy; LSGFD, Laparoscopic sleeve gastrectomy with fundoplication; GERD, gastroesophageal reflux disease.

## Discussion

The incidence of GERD in patients who are obese is higher than that of the general population. The prevalence of GERD in patients who are obese and are considered for bariatric surgery ranges from 50% to 73% (5, 14–16). The most commonly performed bariatric procedure in the world is LSG and several studies have already published the medium and long-term results which demonstrate positive effects with regard to weight loss and comorbidity resolution (17, 18). However, LSG plays an adverse role on the outcomes of GERD (19). In a meta-analysis, Oor et al. (20) reviewed 33 studies, of which 30 studies reported the effect of LSG on the prevalence of GERD symptoms, 12 reported a decrease in the postoperative prevalence of GERD symptoms, and a total of 24 studies reported the incidence of new-onset GERD symptoms, The incidence of *de novo* GERD following LSG can be up to 35% and new-onset esophagitis ranged from 6.3% to 63.3%.These series have raised significant concern and debate around the effect of LSG on GERD. The contributive factors to GERD include a decrease in low esophageal sphincter(LES) pressure (21), esophageal motility dysfunction, injury to the anti-reflux barrier (disruption of the angle of His and division of sling fibers) (22), increased number of transient LES relaxations, reduction in the compliance of the gastric (5), and increased gastric pressure (23). There are still concerns regarding the real effects of LSG on GERD, while Roux-en-Y gastric bypass has demonstrated a postoperative reduction of GERD. Therefore, to treatment and prevention the occurrence or aggravation of GERD after surgery, our research team made an innovative design LSGFD in 2014, which was confirmed by the animal experiments and achieved significant weight-loss and anti-reflux effects.

We chose to perform a LSGFD procedure and aimed to combine the weight loss effect of bariatric surgery with the anti-reflux effect of fundoplication to relieve GERD, or prevent the occurrence of *de novo* GERD, after LSG. Our study examined the results of 45 patients who underwent LSG or LSGFD and monitored the patients for an average of 34 months postoperatively to determine their GERD symptoms and body weight. This study demonstrated that both procedures resulted in a decrease in body weight and there was no statistically significant difference between the two procedures. In addition, this data showed that a significant resolution of GERD after LSGFD and that there was a higher incidence of *de novo* GERD after LSG，based on the GERD-Q score. Several studies have examined the short-term effects of SG combined with fundoplication (sleeve-F) surgery (22). Olmi et al. (12) reported data for 40 patients affected by morbid obesity and GERD who concurrently underwent LSG - Rossetti fundoplication (R-sleeve) and showed good results after follow-up of 12 months. However, one patient experienced food bolus. The BMI and %EWL were 31.2Kg/m^2^ and 61.7%, respectively, and 95% of the patients were without GERD symptoms. In 2020, 220 patients with obesity underwent LSG and a modified Rossetti anti-reflux fundoplication procedure with good postoperative weight loss results and improvement in GERD (13). Our study the retained gastric fundus should not be made too large, which may ensure a good weight loss effect by removing more gastric tissue and we believe that the folded part may has little or no contact with food and has little effect on the secretion of ghrelin hormone. LSGFD with good short-term and medium-term results in weight loss and GERD resolution, while the longer term follow-up result needs a further observation in GERD and weight loss outcomes.

Anatomic changes associated with the LSG procedure may exacerbate GERD symptoms or induce GERD in previously asymptomatic patients. The LSGFD procedure preserves the angle of His and fundoplicatian raises the pressure of the LES to reduce postoperative GERD, thereby treating and/or reducing GERD in patients with a preoperative diagnosis. Our results show that LSG and LSGFD are feasible and safe and no intraoperative complications were reported however, we had one case of leak following LSGFD who recovered after 2 weeks of conservative treatment, other minor complications were cured after symptomatic treatment before discharge. There are several innovations in our procedure: 1) We used a self-made “S”- shaped thick iron wire liver lobe retractor to aid in complete exploration of the lesser curvature of the stomach, especially around the esophageal cardia; 2) we performed a fundoplication depending on the length of the preserved fundus after SG, which may ensure a good weight loss effect by removing more gastric tissue and avoid resection of gastric fold tissue than R-sleeve procedure. In our study, postoperative weight loss was satisfactory with a higher EWL% following LSGFD than LSG. There is no statistically significant difference in weight loss effect between these two procedures. The best weight loss effect was observed at the 12-month follow-up in both procedures. A resolution of GERD symptoms was reported in 86.4% and 90.9% of patients, respectively, at 12 months and the mean 34 (22–48) months after LSGFD. The incidence of *de novo* GERD after LSG was 52.2% at the 12 month and 30.4% at the mean 34 (22–48) months follow up.

Our study had some limitations. The retrospective single center study design had a small population of only 45 patients may be led to selective bias. The postoperative GERD-related results were only based on the GERD-Q score and there was a lack of stronger evidence. Future studies may utilize an objective data assessment with a 24-hour pH-impedance study. It is considered short term for bariatric surgery, while the longer term follow-up result needs a further observation in GERD and weight loss outcomes.

In conclusion, LSG is the most commonly performed bariatric surgical procedure and has a good impact on postoperative weight loss and obesity related morbidities. The effect of LSG on GERD is controversial and LSG is associated with higher rate of postoperative GERD. Despite several limitations, this study highlights that the LSGFD is a feasible and safe procedure in patients who are morbidly obese with GERD, as it has good postoperative results. The incidence of *de novo* GERD after LSG is high and surgeons should evaluate the GERD cautiously before the surgery. LFDSG has a good clinical effect in the treatment of obesity combined with GERD, and LFDSG prevent the occurrence and development of GERD. Future studies should utilize objective assessments to create stronger evidence and make use of a prospective design.

## Data availability statement

The original contributions presented in the study are included in the article/supplementary material. Further inquiries can be directed to the corresponding author.

## Ethics statement

This study involved human participants and was reviewed and approved by the Independent Ethics Committee of People's Hospital of Xinjiang Uygur Autonomous Region December 2017 (NO.2017-94-XJS). We have received patients consent for participation in this study (operation consent, new technology consent etc.).

## Author contributions

AA and MM drafted the manuscript and revised the final version. PM, YT, XL and JC contributed to the investigation and interpretation of the literature. KA revised the final draft. All authors contributed to the article and approved the submitted version.

## Funding

This study was funded by the Research Center of the National Health Commission of Medical and Health Science and Technology Development of China (No. WA2020RW25).

## Acknowledgments

We would like to thank editage (https://www.editage.cn) for language editing.

## Conflict of interest

The authors declare that the research was conducted in the absence of any commercial or financial relationships that could be construed as a potential conflict of interest.

## Publisher’s note

All claims expressed in this article are solely those of the authors and do not necessarily represent those of their affiliated organizations, or those of the publisher, the editors and the reviewers. Any product that may be evaluated in this article, or claim that may be made by its manufacturer, is not guaranteed or endorsed by the publisher.

## Abbreviations

EWL, excess weight loss; GERD, gastroesophageal reflux disease; GERD-Q, gastroesophageal reflux disease questionnaire; LSG, laparoscopic sleeve gastrectomy; LSGFD, laparoscopic sleeve gastrectomy combined with fundoplication; SG, sleeve gastrectomy; TWL, total weight loss.
